# P-1488. Clinical Efficacy of Minocycline in the Treatment of Carbapenem-resistant *Acinetobacter baumannii* Pneumonia

**DOI:** 10.1093/ofid/ofae631.1658

**Published:** 2025-01-29

**Authors:** Hyeri Seok, Si-Ho Kim, Hye Jin Shi, Kyungmin Huh

**Affiliations:** Korea University Medicine, Ansan, Kyonggi-do, Republic of Korea; Division of Infectious Diseases, Samsung Changwon Hospital, Sungkyunkwan University, Changwon, Kyongsang-namdo, Republic of Korea; Division of Infectious Diseases, Department of Internal Medicine, Gil Medical Center, Gachon University College of Medicine, Incheon, Republic of Korea, incheon, Inch'on-jikhalsi, Republic of Korea; Samsung Medical Center, Sungkyunkwan University School of Medicine, Seoultukpyolsi, Seoul-t'ukpyolsi, Republic of Korea

## Abstract

**Background:**

Minocycline is one of the few antibiotics that remain active against carbapenem-resistant *Acinetobacter baumannii* (CRAB). However, the efficacy of minocycline in CRAB pneumonia has not been clearly demonstrated.

**Figure:**
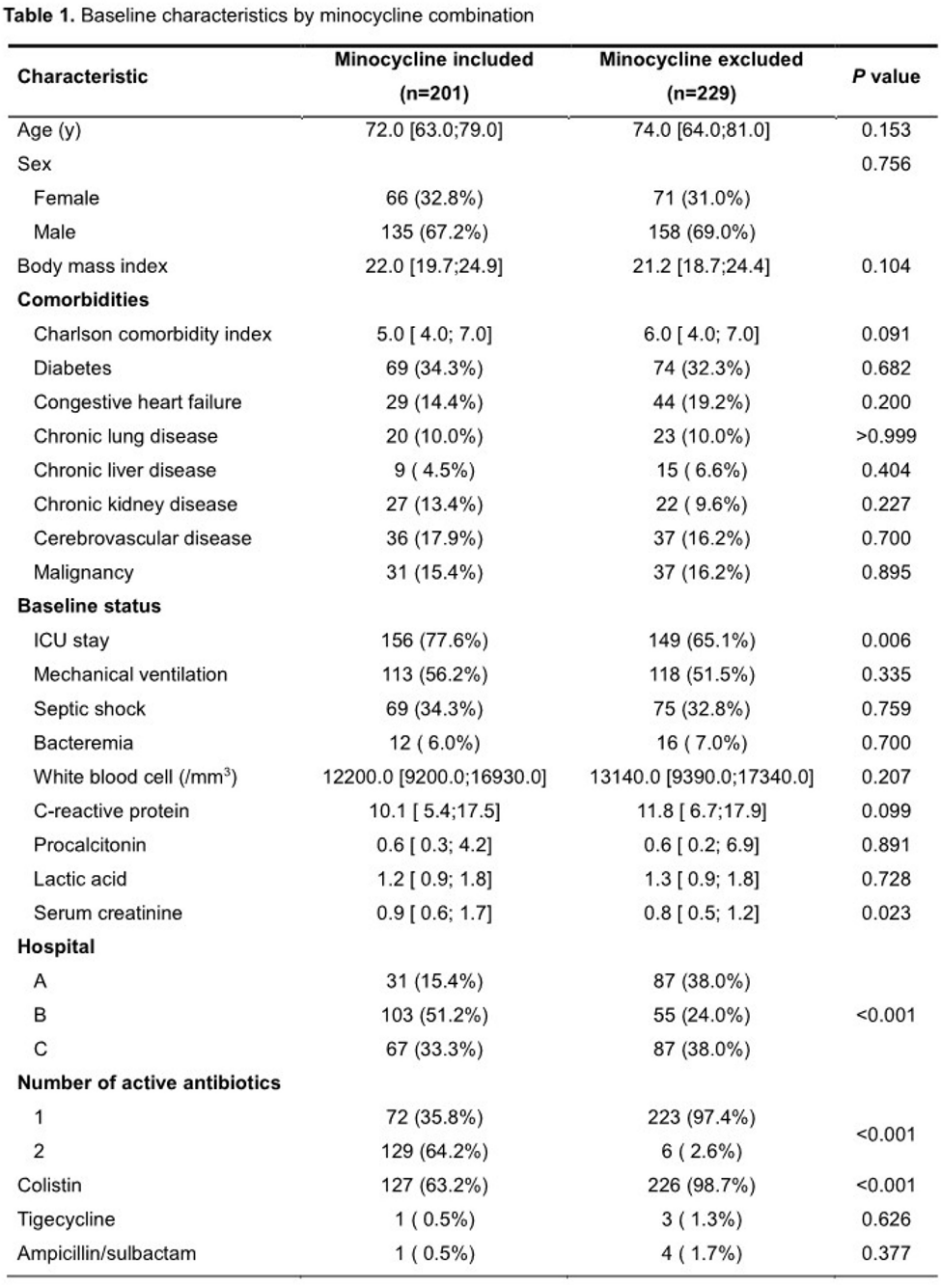

**Methods:**

We conducted a multicenter retrospective cohort study at three university-affiliated teaching hospitals in patients with CRAB pneumonia who were admitted between October 2017 and October 2022. Patients were divided into two groups depending on whether minocycline was included in the antibiotics treatment regimen. Propensity scores for minocycline use were calculated using logistic regression, and logistic regression with overlap weighting was conducted to assess whether there were significant differences in outcome variables and safety variables between the two groups.

**Figure:**
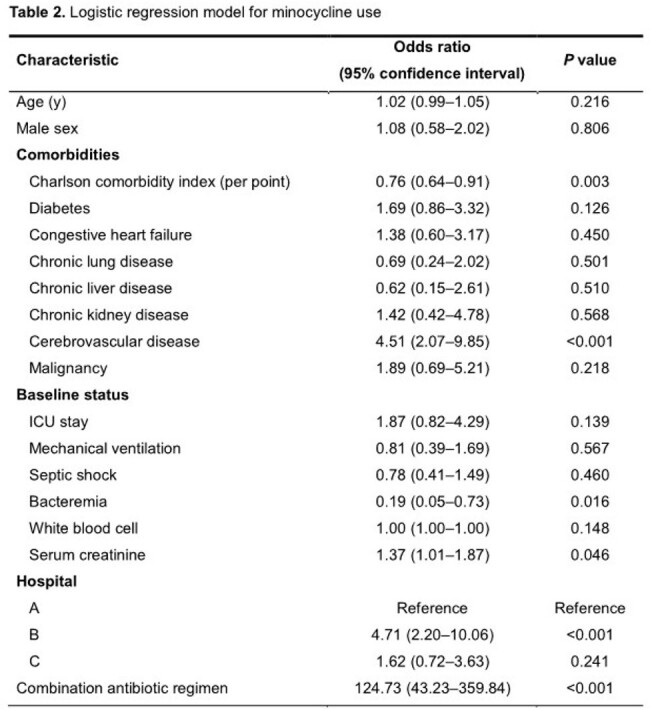

**Results:**

We enrolled 430 CRAB pneumonia patients, among which 201 patients were treated with minocycline and 229 patients without minocycline. Intensive care unit stay was more frequent (77.6% vs 65.1%, *P* value = 0.006) and serum creatinine was higher (0.9 vs 0.8 mg/dL, *P* = 0.023) in the minocycline group than in the non-minocycline group. Variables associated with minocycline use in logistic regression were cerebrovascular disease (odds ratio [OR] 4.51, 95% confidence interval [CI] 2.07–9.85, *P* < 0.001), higher serum creatinine (OR 1.37, 95% CI 1.01–1.87, *P* = 0.046), and combination antibiotic regimen (OR 124.73, 95% CI 43.23–359.84, *P* < 0.001). Bacteremia (OR 0.19, 95% CI 0.05–0.73, *P* = 0.016) and lower Charlson comorbidity index (CCI) (OR 0.76, 95% CI 0.64–0.91, *P* = 0.003) were inversely associated with minocycline use. The clinical success rate on day 14 was higher in the minocycline-treated group (58.3% vs 41.7%, *P* < 0.001). In the adjusted model, the use of minocycline was significantly associated with clinical success (adjusted OR [aOR] 3.88, 95% CI 1.55–9.70, *P* = 0.004), lower 28-day mortality (aOR 0.28, 95% CI 0.10–0.79, *P* = 0.016), and a longer mechanical ventilator-free day (4.51 days longer, 95% CI 2.64–6.37, *P* < 0.001).

**Figure:**
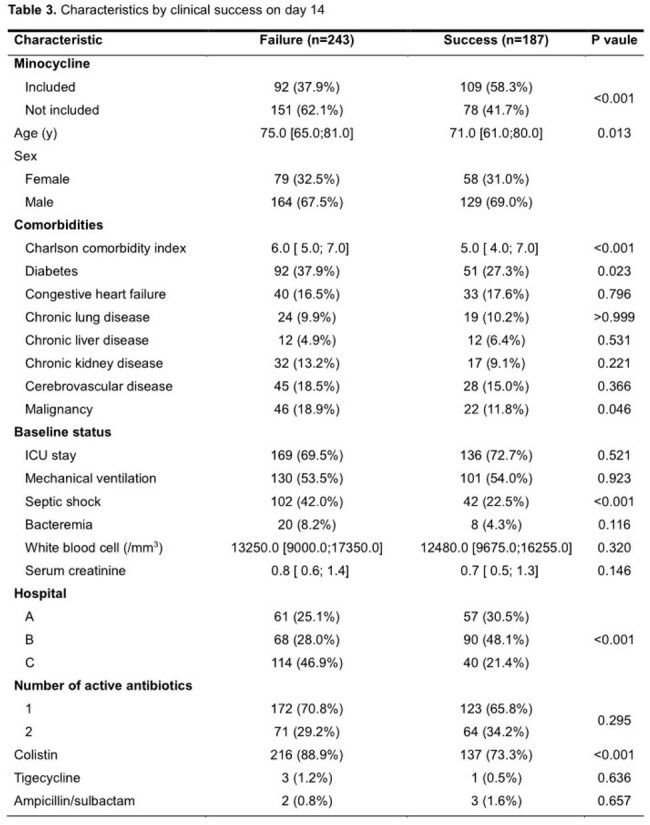

**Conclusion:**

Minocycline, when used as a combination treatment in the treatment of CRAB pneumonia, showed superior results in terms of 14-day clinical success and 28-day mortality. Minocycline may be considered one of the antibiotic treatment options for CRAB pneumonia.

**Figure:**
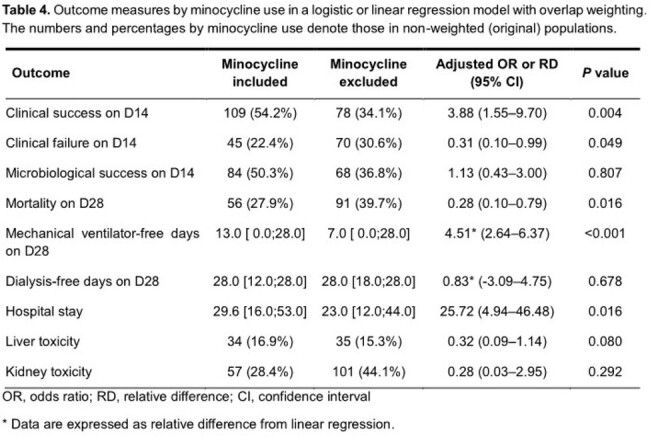

**Disclosures:**

**Kyungmin Huh, M.D., Ph.D.**, bioMérieux: Grant/Research Support|GSK Korea: Advisor/Consultant|Pfizer Korea: Honoraria|Takeda Korea: Advisor/Consultant

